# Auto-antibody testing for children on biologic therapies for rheumatological conditions: results of audit

**DOI:** 10.1186/1546-0096-9-S1-P199

**Published:** 2011-09-14

**Authors:** Julie Duncan, Jill Heath, Eileen Baildam, Gavin Cleary, Michael Beresford, Liza J McCann

**Affiliations:** 1 Alder Hey Children’s NHS Foundation Trust, UK

## Background

Biologic agents may cause auto-antibody formation and drug induced lupus but no paediatric guidelines exist regarding monitoring of auto-antibodies. The UK Royal College of Nursing Guidance advises checking ANA and anti-dsDNA before starting biologic treatment and repeating anti-dsDNA if concerned.

The Alder Hey protocol tested an autoantibody profile (Table 1, 2) 3-6 monthly for data entry for the British Paediatric and Adolescent Rheumatology (BSPAR) Biologics and New Drugs Registry (BDNR) and children with day ward IV biologic administration often had automatic antibodies testing 4-6 weekly.

**Table 1:** 

Diagnosis of patients receiving biologic therapies	N^o^ of Patients
JIA	102
SLE	1
JDM	2
Scleroderma	2
Uveitis (without arthritis)	2
Systemic sclerosis	1
Sarcoid vasculitis	1

**Table 2:** 

Antibody	N^o^ of children with positive results / N^o^ children with antibodies checked [%]	N^o^ of positive antibodies / N^o^ antibodies checked [%]	Comments
ANA	80 / 111 [72%]	431 / 540 [79%]	36 children [32%] ANA always +ve; 44 children ANA sometimes +ve but not always.
Anti ENA	4 / 109 [<4%]	18 / 501 [<4%]	Always non-specific. Negative on subsequent testing.
Anti dsDNA	1/ 111 [<1%]	2 / 510 [<1%]	Negative on subsequent testing.
ANCA	14 / 41 [34%]	25 / 66 [38%]	Always non-specific (non PR3 / MPO).
Anticardiolipin	6 / 98 [6%]	8 / 894 [<1%]	Negative on subsequent testing.

## Aim

To analyse frequency of antibody positivity in rheumatology patients on biologics in order to estimate costs and to devise a rational protocol for test frequency.

## Method

A retrospective audit of a 2 year period, August 2008 to July 2010. All patients on biologics were included with data on auto-antibodies collated using the hospital computer system.

## Results

111 children were receiving biologic therapy and 2511 auto-antibodies were tested at a cost of over £26,000.

## Conclusion

With the exception of ANA antibody, all other antibody tests were usually negative. Those that were positive tended to be non specific (ie. ANCA non-PR3 / MPO; ENA no specific specificity) and/or were negative on subsequent testing. The results did not alter patient care yet costs are significant. This audit would suggest that antibody tests are checked too frequently at Alder Hey Hospital. A new protocol in development is likely to recommend testing auto-antibodies annually in the absence of clinical signs / symptoms of lupus.

